# Influence of Luting Materials on the Retention of Cemented Implant-Supported Crowns: An In Vitro Study

**DOI:** 10.3390/ma11101853

**Published:** 2018-09-28

**Authors:** Ella A. Naumova, Felix Roth, Berit Geis, Christine Baulig, Wolfgang H. Arnold, Andree Piwowarczyk

**Affiliations:** 1 Department of Biological and Material Sciences in Dentistry, School of Dentistry, Faculty of Health, Witten/Herdecke University, Alfred-Herrhausen-Strasse 44, 58455 Witten, Germany; Wolfgang.Arnold@uni-wh.de; 2 Department of Prosthodontics and Dental Technology, School of Dentistry, Faculty of Health, Witten/Herdecke University, Alfred-Herrhausen-Strasse 44, 58455 Witten, Germany; Felix.Roth@uni-wh.de (F.R.); Andree.Piwowarczyk@uni-wh.de (A.P.); 3 Institute for Medical Biometry and Epidemiology, Witten/Herdecke University, Alfred-Herrhausen-Strasse 50, 58455 Witten, Germany; Berit.Geis@uni-wh.de (B.G.); Christine.Baulig@uni-wh.de (C.B.)

**Keywords:** luting materials, implant-supported cobalt–chromium crowns, hydrothermal stress, recementation, retention force

## Abstract

The retention force of cemented crowns on implant abutments with various luting materials was evaluated. Cobalt–chromium crowns were cemented onto tapered titanium abutments (Camlog) with eugenol-free temporary cement (RelyX TempBond NE), composite-based temporary cement (Bifix Temp), zinc phosphate cement (Harvard Cement), glass-ionomer cements (Meron, Fuji I), and resin-modified glass-ionomer cements (Fuji II, Fuji Plus, Ketac Cem Plus). Specimen aging via hydrostress was performed in artificial saliva at 37 °C for 14 days (S1), followed by hydrothermal stress with thermocycling (S2). The crowns were removed, and the force was recorded (T1). Subsequently, the crowns were recemented, aged, and removed, and the force was recorded (T2, T3). The retention forces differences were statistically significant according to the storage conditions at T1 (*p* = 0.002) and T3 (*p* = 0.0002). After aging (S1), Ketac Cem Plus had the highest retention force median value difference (T3 versus T1) (−773 N), whereas RelyX TempBond NE had the lowest (−146 N). After aging (S2), Meron had the highest retention force median value difference (−783 N), whereas RelyX TempBond NE had the lowest (−168 N). Recementation decreased the retention force of the implant-supported cobalt–chromium crowns cemented and recemented with the same luting materials. Luting materials (at T1) and aging conditions significantly impacted the retention force.

## 1. Introduction

Oral dental implant science encompasses numerous topics of interest and evolving thematic trends in clinical studies [1]. Since the 2000s, the focus of dental implant treatment has been biological-driven therapy that recovers and maintains the function, long-term stability, and aesthetics of soft and hard peri-implant tissues [2,3]. Knowledge of the factors that influence the soft and hard peri-implant tissues’ long-term safety and stability has crucial clinical relevance and significance. These factors can be divided into three groups: clinical [4], biological, and technical [4,5]. The biological factors are age, systemic health, medication, oral disease [4], bone support [6], occlusion [7], hard and soft peri-implant tissue quality [4], changes [8] in bacterial colonization around implants and around teeth, type of the oral hygiene [3,9,10], and smoking [4]. The technical factors include amount of retention [11], devices for implant-supported prosthesis retention [12], type of retention force applied [13], crown and implant abutment materials, geometry, height, type of the surface finishing, surface roughness [14,15,16], cleaning method during recementation [17], and the chemical, physical, bioactive and “remove-on-demand” properties of the luting material [11,12,16]. One of the actual topics of interest in the field of biology-driven implant therapy is implant restoration [2,18,19], and this topic has induced the development of new methods and luting materials for implant-supported prosthesis retention [11,20].

There are various reasons for implant restoration. During the application of pressure on the crown occlusal surface, complications such as chipping of the alveolar bone or peri-implantitis can occur, and these complications can require the retrieval of the crowns [21]. As a consequence of the disconnection of the crown, abutment and implant damage can occur and may increase the loss of the implant [22]. Three types of retention of fixed dental implant-supported prosthesis-like crowns are described in the literature: screw retention, cement retention [6,12,23,24], and a combination of both [25,26]. Each type of retention has advantages and disadvantages. Screw-retained restorations are easily retrievable [27] and have fewer technical and biologic complications overall but are expensive [7]. Cemented implant-supported prostheses with a screw access hole in the metal framework improve the survival rates over time and lower the costs of implant-supported prostheses [26]. There are complications, such as disconnection of cement-retained implant-supported prostheses from abutments; nevertheless, this retention mode is still used because it is an effective option, especially for implant-supported single crowns and short-span fixed dental prostheses [18], and because of esthetic and economic reasons [28]. There is a need for the development of new modified luting materials for implant-supported prosthesis retention [11]. Three luting agent types for cement retention are known: temporary, permanent, and semipermanent [11,29,30]. Temporary cements, with low tensile strength and high solubility, help to avoid damaging the restoration and peri-implant tissues [31,32]. Permanent cements with high tensile strength and low solubility induce the opposite mechanical and clinical effects [33]. Semipermanent retention provides adequate retention and retrievability [29]. For semipermanent retention cements, phase change materials (PCM) were created with a phase transition behavior (solid–liquid) depending on temperature or other physical factors [11,29,30]. The matrix of the conventional permanent cement can be changed with activatable microadditives and can acquire new mechanical “remove-on-demand” properties [11,29,30].

Information regarding the retention forces is very important for implant restoration protocols [2]. The aim of this in vitro study was to evaluate the influence of various luting materials after differential artificial aging on the retention of implant-supported cobalt–chromium crowns cemented and recemented with the same luting materials. The primary aim of this in vitro study was the comparison of the required retention forces of the various luting materials under two different aging conditions at three retention measurement time-points. The secondary aim was the evaluation of the influence of the luting materials and aging conditions on the retention force for all retention measurement time-points (T1–T3). The null hypothesis of this in vitro study was that there are no differences between the mean retention forces of the various luting materials at three retention measurement time-points of the implant-supported cobalt–chromium crowns cemented and recemented with the same luting materials.

## 2. Materials and Methods

### 2.1. Preparation of Test Bodies

**Figure 1:**
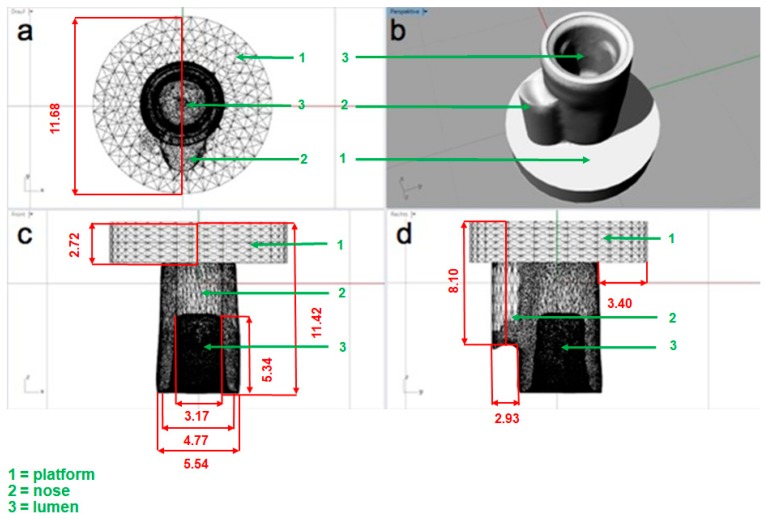
View of the design using the Dental-Designer software (3 shape, Copenhagen, Denmark) with above (**a**), transverse (**b**), and sidelong (**c**,**d**) views of the crown. All sizes are in mm.

Camlog logfit abutments (6° converging angle, 4.3 mm diameter, 5.8 mm height) were screwed with Camlog model implants (Altatec, Wimsheim, Germany). One Camlog logfit abutment (LOT 000034680) was scanned with a model scanner D800 (3 Shape, Copenhagen, Denmark), and a stereolithographic (STL) file was produced. Using dental designer software (18.1) (3 Shape, Copenhagen, Denmark), the STL file of the abutment was used for the crown design for the subsequent standardized decementation (Figure 1).

### 2.2. Cobalt–Chromium Crowns and Luting Materials

Cobalt–chromium crowns were produced by using laser-sintering from a cobalt–chromium alloy (Compartis Co-Cr; Degudent, Hanau, Germany). Cobalt–chromium crowns (*n* = 128) were randomly divided into eight groups (*n* = 16) for standardized cementation onto the corresponding abutments with the following luting materials: one eugenol-free temporary RelyX TempBond NE cement (3M Oral Care, Seefeld, Germany); one composite-based temporary Bifix Temp cement (Voco, Cuxhaven, Germany); one zinc phosphate Harvard cement (Hoffmann Dental, Hoppegarten, Germany); two glass-ionomer cements, i.e., Meron (Voco, Cuxhaven, Germany) and Fuji I (GC, Tokyo, Japan); three resin-modified glass-ionomer cements, i.e., Fuji II (GC, Tokyo, Japan), Fuji Plus (GC, Tokyo, Japan), and Ketac Cem Plus (3M Oral Care, Seefeld, Germany) (Table 1). The cobalt–chromium crowns in all eight groups (*n* = 16) were prepared using standardized cementing with a weight of 6 kg on the abutments.

### 2.3. Artificial Aging after Hydro- and Hydrothermal Stress

To simulate the oral cavity medium and its impact on the luting characteristics of the tested materials immediately after cementation, all specimens were divided according to storage conditions into two groups (S1 and S2): one-half of the specimens (S1) (*n* = 8 for every material group) were subjected to hydrostress (HS) induced by storage at 37 °C for 14 days in 100 mL of artificial saliva (Dental center, Erfurt, Germany) [16]. The other half of the specimens (S2) (*n* = 8 for every material group) were subjected first to hydrostress (HS) and then to hydrothermal stress (HTS), accomplished by thermocycling in a Thermocycler THE1000 (SD Mechatronics, Feldkirchen Westerham, Germany) with 5000 cycles in water baths at temperatures of 5 °C and 55 °C (resistance time 30 s, dripping time 15 s), followed by evaluation of the retention force of the cemented crowns on the implant abutments with the various luting materials.

### 2.4. Retention Force Measurement

After HS (group S1) or HS and following HTS (group S2) using the universal testing machine Texture Analyse HD (Stable Micro Systems, Goldaming, UK), a pull-off test for removing the cobalt–chromium crowns from the abutments at a constant speed of 1 mm/min was performed. The maximum force of the cement failure load in Newtons (N) was recorded (first retention measurement time-point (T1)).

The blasting agent used for sandblasting with the Basic quattro IS (Renfert, Hilzingen, Germany) was aluminum oxide Al_2_O_3_ (Orbis, Muenster, Germany) with a particle size of 50 μm and pressure of 1.0 bar. The plate or the model analogue was manually fixed on the bottom of the blasting basket and blasted at a 45° angle at a distance of 3 cm for approximately 10 s. These values were constantly checked with a set square. After sandblasting, the cobalt–chromium crowns were cemented again with the same luting material, aged by HS (group S1) or by HS and HTS (group S2), and then removed. The cementation–aging–removing cycle for every crown-abutment pair was repeated a total of three retention measurement time-points (T1–T3); for these three retention time-points (T1–T3), the maximum force of the cement failure load (N) was recorded.

### 2.5. Statistics

To evaluate the primary hypothesis, a one-way ANOVA with repeated measures was used at a 5% significance level. Data were described according to the scale level, i.e., medians and quartiles for continuous variables. Multivariate linear regression models were fitted for the target variable retention force to evaluate the impact of the luting material and aging condition at each retention measurement time-point; the results of the fitted models are presented by regression coefficients with related standard errors and 95% confidence intervals as well as results of the Wald test.

The statistical software package SPSS (Statistical Package for Social Sciences, IBM, Armonk, NY, USA) Vers. 23 and R (R Core Team, Vienna, Austria) Vers. 3.3.2 were used.

## 3. Results

### 3.1. Primary Analysis

A repeated measures ANOVA with a Greenhouse–Geisser correction determined that the mean retention forces differed significantly between measurements: F (1.43, 160.44) = 2816.40, *p* < 0.001. Bonferroni-adjusted pairwise comparisons revealed significant differences in retention forces at each retention measurement time-point (Table 2).

Between-subject significant differences of luting materials and storage conditions were detected, *p* < 0.001. Since each within-subject and between-subject effect was statistically significant, multivariate regression analyses were used to investigate the impact of all luting materials and storage conditions on the retention force at T1, T2, and T3, separately. Harvard was treated as the reference material.

At T1, RelyX Temp Bond NE, Meron, Fuji I, Fuji II, and Bifix Temp showed significant increases or decreases in the retention force in comparison to Harvard at a local 5% significance level; hydrothermal stress caused a significant decrease in comparison to hydrostress (Table 3). 

At T2, RelyX Temp Bond NE, Meron, Bifix Temp, and Ketac Cem Plus showed significant decreases in the retention force in comparison to Harvard at a local 5% significance level; hydrothermal stress caused a significant decrease in comparison to hydrostress (Table 4).

At T3, RelyX Temp Bond NE, Bifix Temp, and Ketac Cem Plus showed significant decreases in the retention force in comparison to Harvard at a local 5% significance level; hydrothermal stress caused a significant decrease in comparison to hydrostress (Table 5).

### 3.2. Comparison of Retention Forces at T1, T2, and T3

**Figure 2:**
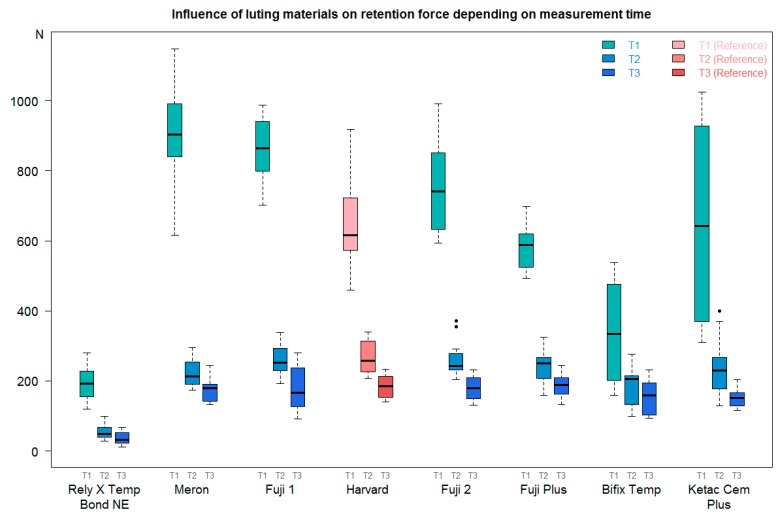
Comparison of the retention force at T1–T3 independent of the storage conditions. Boxes indicate the data’s location and variation. One box includes 50% of the analyzed data; the line within the box indicates the median.

At T1, the required retention force was considerably higher than at T2 or T3 (Table 6). In general, the retention force was continuously reduced from T1 to T3. The median retention force at T1 (independent of luting material or storage condition) was 619.20 N, with an interquartile range (IQR) of 410.40–852.20 N. At T2, the median force decreased to 226.70 N (IQR 190.00–259.20 N); therefore, the lowest force, corresponding to 162.80 N (IQR 132.30–198.60 N), was reached at T3 (Figure 2).

### 3.3. Comparison of Retention Forces at T1, T2, and T3 for Luting Materials Independent of the Storage Conditions

**Figure 3:**
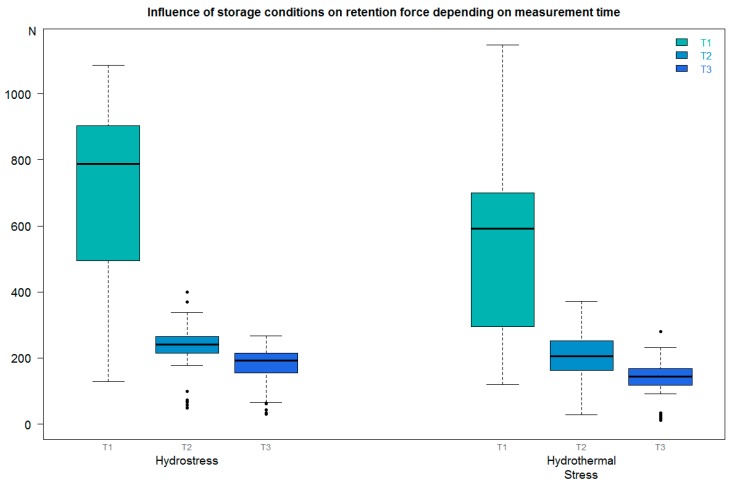
Comparison of the retention force at three retention measurement time-points T1–T3 after various storage conditions independent of the luting materials. For a description of the boxplot, see Figure 2.

At T1, the luting material RelyX Temp Bond NE achieved the minimum retention force required (Table 7, Figure 3). Temp Bond NE showed the lowest median retention force (191.70 N; IQR 155.60–224.39 N), while Meron showed the highest (902.30 N; IQR 848.40–973.90 N). Ketac Cem Plus had the highest standard deviation of the mean retention force (±295.46 N).

At T2, RelyX Temp Bond NE had the lowest median retention force (49.09 N; IQR 38.89–65.89 N), and Harvard had the highest (258.10 N; IQR 225.70–308.70 N). Ketac Cem Plus reached the highest maximum retention force (399.30 N). The results are shown in Table 8 and Figure 3.

The lowest median retention force at T3 was achieved by RelyX Temp Bond NE (30.98 N; IQR 22.63–47.93 N), and the highest median retention force was achieved by Fuji Plus (188.60 N; IQR 164.90–209.10 N). The results can be found in Table 9 and Figure 3.

### 3.4. Comparisons of Retention Forces at T1, T2, and T3 for Storage Conditions Independent of Luting Materials

At each retention measurement time-point (T1, T2, and T3), the retention force after storage under hydrostress was conspicuously higher than after storage under hydrothermal stress (Figure 3, Table 10, Table 11 and Table 12).

## 4. Discussion

The success rates after dental implant treatment are high [23,34,35], and the need for future removal and reparation of implant-fixed restorations will increase [11]. The choice of cement for implant-fixed restorations can influence implant stability after restoration removal.

The retention of crowns on abutments is achieved by a combination of height and form of an abutment, surface roughness of crown and abutment, the luting material [29,36], aging, and the number of recementations. The present in vitro study was performed to observe only three of these factors: the influence of the luting material types, storage conditions (aging), and number of recementations. All other parameters, such as materials, size, and form of the abutment and crown, were standardized. This is why the surface roughness of the crown and abutment were not measured. Titanium abutments were chosen for the present in vitro study because the material of the abutments influences the accumulation of bacteria on the abutments [37] as well as the quality and quantity of marginal soft tissue [14]. Abrahamsson et al. [14] observed that titanium increased new soft tissue formation. Cobalt–chromium crowns were investigated because they are low-cost and have good corrosion resistance and higher hardness [30]. However, cobalt–chromium crowns exhibit lower detail accuracy and higher shrinkage after casting than gold alloys [30]. The superstructure geometry and related surface peculiarities influenced the mechanical behavior of the various abutment–prosthesis (crown) connections [38,39]. A conical connection between abutment and crown was performed because in vivo studies revealed significantly lower bacterial counts in the peri-implant sulci and inside the connections in the internal hexagons of the external collar connection and conical connection groups [4,40]. Clinical experience was considered in the selection of the luting materials for the present study [28], which were: the commonly used conventional permanent zinc phosphate and the glass ionomer cements [41], popular in the last decade, permanent resin-modified glass ionomer cements [41], and temporary cements [31]. The permanent zinc phosphate Harvard cement served as a control, the “gold standard for comparisons with all other luting agents”; we are grateful for its long and successful clinical history [41]. A sample size calculation was not carried out. Per se *n* = 8 samples per cement group and storage conditions were prepared. This number of cases was found sufficient in previous studies for cement adhesion tests and retention tests of temporary or permanent luting agents for implant-retained dentures [42,43]. A post-hoc power analysis confirmed the investigation’s sample size choice of *n* = 8 per storage condition and luting material as sufficient. The retention forces of all luting materials at T1 were compared to that of the reference material (Harvard cement) for each storage condition separately, and when taking into account the problem of multiple testing by usage of the Holm–Boferroni method. It could be shown that the pairwise comparisons had a post-hoc effect power ranging between 84% and 99%. The aging of the tested specimens for retention force evaluation was performed with HS and HTS that made the present study comparable to similar investigations [27,44,45,46]. Data from the present study clearly demonstrated that the bonding capacities of the various luting materials had a remarkable influence on the retention force only after the first cementation. The highest retention force was achieved by the permanent glass ionomer cement Meron; however, Kunt et al. [27] reported that Meron had a more than three times lower retention force (pull-off force) than that measured in the present study and a lower pull-off force compared to the permanent zinc oxide phosphate cement Adhesor. The variation of these results can be explained by the lower pull rate (0.5 mm/min versus 1.0 mm/min in the present study), the use of another metal for the crown, the smaller size (diameter × height) of the abutment (3.7 mm × 5.0 mm versus 4.8 mm × 5.3 mm in the present study), and the conventional casting process for the crown production (versus the laser-sintered process in the present study). Kilicarslan and Ozkan [47] found significantly higher pull-off forces for laser-sintered superstructures on implant abutments compared to those of conventional castings. The eugenol-free temporary Rely X Temp Bond NE showed the lowest retention force. This finding agrees with those of other studies [27,44]. Michalakis et al. [44] found a significantly lower retention force of eugenol-free temporary luting materials than for temporary composite-based cement Bifix Temp. Kunt et al. [27] also showed the lowest retention force for the eugenol-free temporary cement Cavex Temporary, which belongs to the same material class as Rely X Temp Bond NE. In the present study, the permanent methacrylate-based resin-modified glass ionomer cements and permanent zinc phosphate cement showed small differences in retention force median values. Korsch et al. [48] and Pesce, Canullo et al. [4] observed that methacrylate cements have low viscosity and can leave more excess cement in the implant–mucosal interface, causing bleeding and inducing biofilm formation [4,48]. With respect to the various storage conditions (artificial aging), only at retention measurement time-point T3 could a locally significant difference of the retention force between groups (S1 and S2) be determined. This finding agrees with the results of a study by Michalakis et al. [44], where all specimens showed a reduction in the maximum retention force (pull-off force) after HTS. However, Dudley et al. [45] showed that the glass ionomer cement (Ketac Cem) and the eugenol-free provisional cement (Temp Bond NE) showed no significant differences in terms of maximum retention force with an increasing number of thermocycles [45], possibly due to the absence of the retentions and recementations between the examinations. With increasing retentions and recementations, the retention (pull-off) force for all test specimens was reduced in the present experiments. Obviously, the characteristics of cemented implant-supported cobalt–chromium crown internal surfaces in the present study were altered after the retention and surface conditioning with sandblasting, significantly reducing the crown–cement–abutment bonding interaction and consequently altering the retention force during the second and third retention in all of the cement groups, independent of the luting materials. Mundt et al. [30] used a similar sandblasting model of the cobalt–chromium crown inner surfaces with 50 mm particle size aluminum oxide at a pressure of 2.5 bar, followed by rinsing and drying, reporting similar observations. However, an in vitro study investigating the retention of implant-supported CAD/CAM metal copings after cementation with temporary cements and recementation with the permanent resin cement Panavia showed that the bond strength of the adhesive resins was not affected after minor surface alterations occurring during recementation [46]. The differences in the results can be explained by different study designs: the initial cementation was performed with temporary cement; however, the recementations were performed with temporary resin cements. In the present study, by contrast, the initial cementation and following recementations were performed with the same luting materials. It appears that the effect of decreasing retention force due to recementation can be altered by using another type of luting material for the subsequent recementations. Perhaps, resin cements are more appropriate for this purpose. In some studies, it was observed that the resin cement Panavia contains 10-methacryloyloxydecyl dihydrogen phosphate (MDP) and provides the highest retentive values due the chemical bond with metallic oxides [46,49].

We rejected the null hypothesis of this in vitro study, i.e., that there are no differences between the mean retention forces of the different luting materials at three retention measurement time-points of the implant-supported cobalt–chromium crowns cemented and recemented with the same luting materials.

The present study has some limitations: only hydro- and hydrothermal stress were performed to induce artificial aging. Unfortunately, this aging is not completely appropriate for the dynamic nature of intraoral aging [49]. Second, surface roughness was not measured. Another limitation was the type of the retention force due decementation. The force within the pull-off test was always applied along the vertical prosthetic axis at a constant speed, and this is almost never possible in clinical practice [12,49]. In future research, the surface roughness before and after every recementation trial should be measured. Furthermore, the alterations of the crown surfaces after multiple recementations should be studied [30]. Most likely, for future research, special alternative methods and condition agents for preserving cobalt–chromium crown internal surface conditions have to be tested, possibly providing an alternative to sandblasting. Additional masticatory simulation in experiments can improve the simulation of intraoral aging, because the nature of these loads are oblique occlusion forces, and the stress state during mechanical fatigue by thermal deformation is different [49] compared to hydro- and hydrothermal stress. Recementation of cemented implant-supported cobalt crowns with other luting materials should be performed. The results of in vitro studies, such as this study, should be included in a database regarding luting material characteristics and should be correctly interpreted. There remains a need for guidelines regarding cement or cementation procedures and for generating accurate information regarding the clinical outcomes of cement-retained implant-supported fixed restorations, particularly regarding the ideal type of cement that would provide stability and maintain retrievability [18], ensuring biological compatibility and simple removal of excess cement using radiographic view [50] and controlled destruction of dental cements [11].

## 5. Conclusions

Sandblasting and recementation of implant-supported cobalt–chromium crowns with the same luting materials resulted in a reduction of the retention force independent of the luting material. Within the limitations of this study, it can be concluded that a material-specific ranking of cemented implant-supported cobalt–chromium crown retention forces was observed due to the first decementation. Clinical research is needed to confirm these findings.

## Figures and Tables

**Table 1:** Description of the luting materials used in this study.

Material	Type	Chemical Composition ^a^	Application	Manufacturer
RelyX TempBond NE	temporary eugenol-free cement	P: zinc oxide L: white mineral Oil (Petroleum)	paste/paste	3M Oral Care, Seefeld, Germany
Meron	permanent glass ionomer cement	P: fluoraluminosilicate glass L: polyacrylic acid	capsule	VOCO, Cuxhaven, Germany
Harvard Cement	permanent zinc phosphate cement	P: zinc oxide, magnesia L: phosphoric acid	powder/liquid	Hoffmann Dental, Hoppegarten, Germany
Fuji I	permanent glass ionomer cement	P: fluoroaluminosilicate glass L: polyacrylic acid	powder/liquid	GJ, Tokyo, Japan
Fuji II	permanent resin-modified glass ionomer cement	P: fluoroaluminosilicate glass L: methacrylated polyacrylic acid	paste/paste syringe	GJ, Tokyo, Japan
Fuji Plus	permanent resin-modified glass ionomer cement	P: fluoraluminosilicate glass L: methacrylated polyacrylic acid	capsule	GJ, Tokyo, Japan
Bifix Temp	temporary composite-based cement	B: triethylene glycol dimethacrylate C: benzolperoxid	paste/paste	VOCO, Cuxhaven, Germany
Ketac Cem Plus	permanent resin-modified glass ionomer cement	P: fluoroaluminosilicate glass L: methacrylated polyacrylic acid	paste/paste syringe	3M Oral Care, Seefeld, Germany

a, according to the information provided by the manufacturers. Abbreviations, P = powder; L = liquid; B = base; C = catalyst.

**Table 2:** Bonferroni-adjusted pairwise comparisons of mean differences in retention force. T1, T2, T3: retention measurement time-points.

Retention Measurement Time-Point		Mean Difference	Standard Error	*p*-Value
T1	T2	401.455	7.768	<0.001
T1	T3	461.429	7.559	<0.001
T2	T3	59.974	4.077	<0.001

**Table 3:** T1: Multivariate linear regression analysis to investigate the impact of all luting materials and storage conditions on the retention force.

Covariable	Regression Coefficient	Standard Error	T	*p*-Value	Lower 95%-CI	Upper 95%-CI
(Intercept)	726.98	33.14	21.94	<0.001	661.37	792.59
RelyX Temp Bond NE	−462.24	44.18	−10.46	<0.001	−549.72	−374.75
Meron	263.04	44.18	5.95	<0.001	175.56	350.53
Fuji I	213.52	44.18	4.83	<0.001	126.03	301.00
Fuji II	101.09	44.18	2.29	0.024	13.61	188.58
Fuji Plus	−68.49	44.18	−1.55	0.124	−155.98	18.99
Bifix Temp	−313.95	44.18	−7.11	0.001	−401.44	−226.47
Ketac Cem Plus	−4.12	44.18	−0.09	0.926	−91.60	83.37
Hydrothermal Stress	−150.82	22.09	−6.83	<0.001	−194.56	−107.07

**Table 4:** T2: Multivariate linear regression analysis to investigate the impact of all luting materials and storage conditions on the retention force.

Covariable	Regression Coefficient	Standard Error	T	*p*-Value	Lower 95%-CI	Upper 95%-CI
(Intercept)	285.62	12.13	23.55	<0.001	261.60	309.63
RelyX Temp Bond NE	−218.62	16.17	−13.52	<0.001	−250.54	−186.59
Meron	−47.57	16.17	−2.94	0.004	−79.59	−15.55
Fuji I	−12.59	16.17	−0.78	0.438	−44.62	19.43
Fuji II	−10.90	16.17	−0.67	0.502	−42.92	21.12
Fuji Plus	−28.16	16.17	−1.74	0.084	−60.19	3.86
Bifix Temp	−84.71	16.17	−5.24	<0.001	−116.73	−52.69
Ketac Cem Plus	−34.25	16.17	−2.12	0.036	−66.28	−2.23
Hydrothermal Stress	−29.59	8.09	−3.66	<0.001	−45.60	−13.58

**Table 5:** T3: Multivariate linear regression analysis to investigate the impact of all luting materials and storage conditions on the retention force.

Covariable	Regression Coefficient	Standard Error	T	*p*-Value	Lower 95%-CI	Upper 95%-CI
(Intercept)	204.36	8.47	24.12	<0.001	187.59	221.14
RelyX Temp Bond NE	−149.63	11.30	−13.25	<0.001	−172.00	−127.26
Meron	−11.95	11.30	−1.06	0.293	−34.32	10.43
Fuji I	−3.17	11.30	−0.28	0.779	−25.54	19.20
Fuji II	−5.30	11.30	−0.47	0.640	−27.67	17.07
Fuji Plus	2.04	11.30	0.18	0.857	−20.33	24.41
Bifix Temp	−29.62	11.30	−2.62	0.010	−51.99	−7.25
Ketac Cem Plus	−34.57	11.30	−3.06	0.003	−56.94	−12.20
Hydrothermal Stress	−38.18	5.65	−6.76	<0.001	−49.37	−27.00

**Table 6:** Descriptive data of the statistical evaluation of the retention force (in N).

Retention Measurement Time-Point	Minimum	1st Quartile	Median	Mean Value	SD	3rd Quartile	Maximum
T1	119.70	410.40	619.20	617.70	273.65	852.20	1148.00
T2	27.28	190.00	226.70	216.20	81.67	259.20	399.30
T3	10.45	132.30	162.80	156.20	59.83	198.60	279.40

**Table 7:** Descriptive data of the statistical evaluation of the retention force at T1 independent of the storage conditions (in N).

Luting Material				T1			
	Minimum	1st Quartile	Median	Mean Value	SD	3rd Quartile	Maximum
RelyX Temp Bond NE	119.70	155.60	191.70	189.30	47.47	224.39	280.90
Meron	615.20	848.30	902.30	914.60	130.90	973.90	1148.00
Fuji I	702.30	800.50	863.60	865.10	87.44	936.20	987.90
Harvard	459.10	575.90	615.80	651.60	129.90	711.30	919.00
Fuji II	593.30	636.60	740.10	752.70	135.13	829.50	991.70
Fuji Plus	492.50	528.40	588.50	583.10	62.66	617.80	698.20
Bifix Temp	158.30	204.00	334.50	337.60	137.92	466.40	538.30
Ketac Cem Plus	310.00	370.80	642.20	647.50	295.46	899.80	1024.00

**Table 8:** Descriptive data of the statistical evaluation of the retention force at T2 independent of the storage conditions (in N).

Luting Material				T2			
	Minimum	1st Quartile	Median	Mean Value	SD	3rd Quartile	Maximum
RelyX Temp Bond NE	27.28	38.89	49.09	52.21	19.11	65.85	98.55
Meron	172.90	190.90	213.60	223.30	36.64	253.90	295.50
Fuji I	192.30	232.20	251.40	258.20	44.51	292.50	338.30
Harvard	206.70	225.70	258.10	270.80	46.68	308.70	340.30
Fuji II	203.20	231.50	242.40	259.90	46.07	272.00	370.60
Fuji Plus	159.40	209.10	249.20	242.70	42.93	265.10	325.30
Bifix Temp	99.47	137.70	205.00	186.10	53.69	214.70	275.90
Ketac Cem Plus	128.90	180.00	229.10	236.60	75.84	255.30	399.30

**Table 9:** Descriptive data of the statistical evaluation of the retention force at T3 independent of the storage conditions (in N).

Luting Material				T3			
	Minimum	1st Quartile	Median	Mean Value	SD	3rd Quartile	Maximum
RelyX Temp Bond NE	10.45	22.63	30.98	35.64	19.15	47.93	66.78
Meron	131.90	142.40	179.30	173.30	31.27	190.10	244.50
Fuji I	91.93	129.40	165.30	182.10	61.05	237.30	279.40
Harvard	140.20	153.50	185.30	185.30	30.48	211.60	234.00
Fuji II	131.20	154.80	178.80	180.00	34.37	207.50	231.70
Fuji Plus	132.80	164.90	188.60	187.30	31.77	209.10	244.20
Bifix Temp	92.92	102.40	158.90	155.70	48.12	194.60	232.40
Ketac Cem Plus	116.20	132.10	150.60	150.70	26.52	164.10	204.10

**Table 10:** Descriptive data of the statistical evaluation of the retention force at T3 independent of the luting materials (in N).

Storage Condition				T1			
	Minimum	1st Quartile	Median	Mean Value	SD	3rd Quartile	Maximum
Hydrostress	128.60	494.90	788.40	693.10	263.76	899.80	1086.00
Hydrothermal stress	119.70	302.70	590.80	542.30	264.25	699.10	1148.00

**Table 11:** Descriptive data of the statistical evaluation of the retention force at T3 independent of the luting materials (in N).

Storage Condition				T2			
	Minimum	1st Quartile	Median	Mean Value	SD	3rd Quartile	Maximum
Hydrostress	47.93	214.30	241.30	231.00	75.11	265.50	399.30
Hydrothermal stress	27.28	162.80	205.00	201.40	85.78	251.20	370.60

**Table 12:** Descriptive data of the statistical evaluation of the retention force at T3 independent of the luting materials (in N).

Storage Condition				T3			
	Minimum	1st Quartile	Median	Mean Value	SD	3rd Quartile	Maximum
Hydrostress	29.16	155.90	191.40	175.30	58.05	214.00	267.50
Hydrothermal stress	10.45	116.80	142.90	137.20	55.74	167.60	279.40
